# (Mis)representing the Opposition and Rhetorical Success: Experimental Evidence on Faithful and Inaccurate Reformulations

**DOI:** 10.1007/s10503-026-09692-5

**Published:** 2026-01-28

**Authors:** Ramy Younis

**Affiliations:** University of Fribourg, Fribourg, Switzerland

**Keywords:** Straw man, Misrepresentation, Steel man, Persuasion, Trustworthiness, Experimental research

## Abstract

Previous research in argumentation has closely examined distortions of the opposition—particularly the straw man—and has recently provided some experimental evidence on their effects on persuasive outcomes. However, comparatively little empirical attention has been given to the inverse practice of faithfully reformulating an opponent’s contribution. The effects of accurate and inaccurate representations on speaker *ethos* and perceived reasonableness also remain underexplored. This paper addresses these gaps through three pre-registered experimental studies comparing *accurate reformulation*, *misrepresentation*, and *no reformulation* of the opposition. Experiment 1 assesses the impact of these practices on perceived trustworthiness using a six-item, 7-point semantic differential scale. Experiment 2 examines judgments of reasonableness using a scale repeatedly employed in pragma-dialectical effectiveness research. Experiment 3 measures persuasiveness at both the attitudinal and behavioral intention levels. Participants read a series of pre-tested argumentative exchanges between two speakers in a charitable-giving context. Results show that, in the cases examined, misrepresenting the opposition negatively impacted both trustworthiness and reasonableness judgments, addressing concerns that adhering to dialectical standards may diminish rhetorical success.

## Introduction

Within argumentation theory and persuasive communication research, a substantial body of work focuses on how speakers represent an opponent’s contributions in argumentative discourse. This research addresses, among other things, normative questions about which representational practices constitute *reasonable* or *fallacious* moves, as well as descriptive and explanatory questions concerning *how* and *why* such representations *affect persuasion*.

A central focus of this work has been the *misrepresentation* or *distortion* of an opponent’s argumentation. Specifically, extensive attention has been devoted to the *straw man*: the practice of misrepresenting the opposition to make it easier to attack. Such studies have been concerned with clarifying the (fallacious) nature of the straw man (Aikin and Casey 2022; Lewiński and Oswald 2013; van Eemeren and Grootendorst 2004; Walton 1996), carefully delineating the different forms it can take (Aikin and Casey 2011, 2013, 2016, 2022), and shedding light on its persuasive effects (Bizer et al. 2009; Oswald and Lewiński 2014; Schumann 2022).

The present research extends this body of work by experimentally investigating the *rhetorical effects* of different representational practices, including but not limited to misrepresentation. Recent work has already begun to adopt experimental approaches to better understand the impact of the straw man on persuasive outcomes (e.g., Bizer et al. 2009; Schumann 2022). However, little empirical attention has been given to the practice of presenting an *accurate* and *clear* reformulation of the opposition before attempting to refute it. Dennett (2013) and Rapoport (1960, 1961) recommend such a practice to avoid common pitfalls, such as caricaturing an opponent, and they point to its potential rhetorical benefits. Nonetheless, its impact remains empirically underexplored. Moreover, the effects of (mis)representation of the opposition on perceived credibility remain largely untested, despite the central role of *ethos* in persuasive communication (see O’Keefe 2016; Petty et al. 1981; Sperber et al. 2010). Finally, while there is extensive experimental work examining how different fallacies affect judgments of reasonableness (van Eemeren et al. 2009; Eemeren et al. 2012), empirical evidence on the specific impact of the straw man fallacy on perceived reasonableness is still lacking.

The present study seeks to address these gaps by experimentally comparing the effects of messages that contain *accurate reformulations*, *misrepresentations* or *no* *reformulation* of the opposition. Three pre-registered experiments in the charitable-giving context (see Caviola et al. 2020; MacAskill 2015; Schubert and Caviola 2024) are presented, each assessing a different dependent variable: perceived trustworthiness, judgments of reasonableness, and persuasive outcomes.

A central concern motivating this study is whether adhering to normative standards in argumentation reduces rhetorical success, a question clearly articulated by O’Keefe (2003, 2006). In other words, the study relates to the broader question of whether, and to what extent, reasonable argumentation and rhetorical effectiveness are in conflict. In the context of the present study, the specific concern is how different ways of re-expressing an opponent’s position relate to rhetorical success. As O’Keefe (2006) highlights, these questions are empirical in nature, and can effectively be addressed through experimental research.

The following section lays the groundwork for the empirical studies presented in this paper by clarifying the distinction at the center of the present investigation, namely the contrast between *misrepresentation* and *accurate reformulation*, and by reviewing prior empirical research relevant to the rhetorical effects of these representational practices.

## Theoretical and Empirical Background: Representing the Opposition

### Misrepresentation and Accurate Reformulation

Within theories of argumentative normativity, misrepresenting the opposition is generally viewed as a violation of dialectical requirements. In the pragma-dialectical theory of argumentation, distorting an opponent’s commitments constitutes a violation of the dialectical norms of *reasonableness*. This approach, grounded in critical rationalism, puts critical discussion at the center of its conception of reasonableness (van Eemeren and Grootendorst 2004, pp. 131–134). Misrepresenting the opposition is thus a normative violation as it hinders the resolution of a difference of opinion in a critical discussion. Specifically, misrepresenting an opponent’s argumentation is considered a fallacious move insofar as it violates one of the rules articulated by van Eemeren and Grootendorst that specify when a contribution hinders or contributes to the resolution of the difference. The rule in question is *Rule 3*,* the Standpoint Rule*:

Attacks on standpoints may not bear on a standpoint that has not actually been put forward by the other party. (2004, p. 191)

The rationale behind the inclusion of this rule is that if an attack is not related to the argumentation^1^ that the target has actually put forward, the parties fundamentally talk at cross-purposes, which in turn stands in the way of achieving a reasonable resolution of the original disagreement (van Eemeren et al., 2002, pp. 116–117; see also Walton 1996).

In this respect, it is important to note that, as Aikin and Casey delineate in their work (2011, 2013, 2016, 2022), distortions of the opposition can take different forms. Perhaps the most widely discussed among these is the *straw man*, which consists in an unfaithful representation that *weakens* the opposition to make it *easier to attack*^2^ (Aikin and Casey 2022; Bizer et al. 2009; Lewiński and Oswald 2013; Schumann et al. 2019; Walton 1996). However, there can also be *charitable* distortions that *improve* another’s position. This latter practice is what Aikin and Casey call the *iron man* (2013). The core insight behind the iron man is that a representation can distort an arguer’s contribution not only by *weakening* their expressed views but also by *strengthening* them.

What iron man and straw man have in common is their funhouse mirror representation of the original contribution. Indeed, the two practices involve, by definition, a distortion of a speaker’s commitments^3^. By contrast, a faithful representation attributes to a speaker the commitments that they have, in fact, taken on, accurately capturing what was communicated.

In their work on the straw man, Lewiński and Oswald (2013) refer to this criterion for interpretation as *pragmatic plausibility*. The standard requires that one follow procedures for a contextually plausible interpretation and thus stay within the confines of a *disagreement space* of an utterance, where disagreement space refers to “all the commitments an arguer may be held accountable for” (2013, p. 169; see also Jackson 1992). This notion is encapsulated by a principle discussed by Govier: “interpret as well as you can”, that is, “do not miss nuances of meaning, irony, humour, sarcasm, qualifying phrases, relevance of preceding and subsequent material, and so on” (1981, p. 5). A pragmatic account of communication can then guide interpretation by offering tools to carefully attribute commitments^4^.

Compared to misrepresentation and the straw man, relatively little research has focused on the practice of presenting a faithful reformulation of the opposition before attempting to refute it. Dennett (2013) recommends such a practice as one of four rules for composing “a successful critical commentary”, drawing from similar principles by game theorist Rapoport^5^ (1960, 1961). The strategy involves providing a clear, vivid, and fair reformulation of one’s opponent’s position that the target would find no fault with (Dennett 2013, p. 33). The author presents this approach as a strategy for avoiding common pitfalls such as caricaturing one’s opponent or nitpicking. It should be noted that this practice is sometimes conflated with overly charitable *distortions* wherein the argumentation is *improved*, often under the label *steel man* (De Rijk 2024; Pruś and Sikora 2023). However, a closer reading reveals that the approach does not require strengthening arguments or standpoints, but only ensuring they are represented fairly and clearly^6^ (2013, p. 33).

Both Rapoport and Dennett highlight a rhetorical benefit to the strategy, noting that following this practice has the effect of showing that one understands an opponent’s position as well as they do, which in turn can positively impact persuasive success (Dennett 2013, p. 33; Rapoport 1961, p. 216).

### Representing the Opposition and Rhetorical Success

The present study is concerned with experimentally comparing the rhetorical effects of messages that contain faithful reformulations of an opponent’s position with those that contain misrepresentations of the opposition. Such an investigation connects to a broader concern that lies at the intersection of argumentation theory and persuasive communication research, namely whether adhering to normative standards comes *at the expense* of persuasive success (see O’Keefe 2003, 2006)^7^. Given the prevalence of fallacies in public discourse and a tendency to view dialectic and rhetoric as rival paradigms^8^, one may worry that reasonable argumentation and rhetorical effectiveness are in fundamental conflict. However, as O’Keefe points out, whether such a tension exists in any given instance is an empirical question for which experimental evidence can be brought to bear (2006).

To start addressing this concern, O’Keefe reviews empirical evidence in connection to three principles for normatively good argumentative practice: (i) speakers should put forward their standpoints in a clear manner, (ii) arguers should present their supporting arguments clearly, and (iii) speakers should defend their standpoints against counterarguments (2006). The author draws these principles from pragma-dialectical rules for a critical discussion (Rule 2 and Rule 10) and investigates how adherence to them affects rhetorical success. As O’Keefe highlights by means of meta-analytic reviews, relevant empirical evidence in the research literature indicates that adhering to these specific norms for reasonable argumentation does not diminish rhetorical success but, if anything, leads to better persuasive outcomes (2006, pp. 236–240).

Relatedly, van Eemeren, Garssen and Meuffels have conducted extensive experimental research investigating whether the standards laypeople apply in judging the reasonableness of argumentative contributions align with theoretically-motivated rules for a critical discussion (2009, 2012). Participants in the experiments were presented with fallacies as well as fragments with no normative violation and were asked to evaluate the *reasonableness* of the contributions. The authors put to the test a variety of fallacies connected to different pragma-dialectical rules (e.g., *ad hominem* as a violation of the freedom rule or *evading the burden of proof* as a violation of the obligation-to-defend rule). Their results provide strong evidence that ordinary arguers evaluate moves that violate normative requirements as clearly less reasonable than non-fallacious moves, supporting the conventional validity of the rules.

There is therefore a valuable body of empirical work shedding light on the relationship between reasonableness and persuasiveness. However, these studies have focused only on a selection of rules and fallacies. Crucially, they do not provide empirical evidence on the perceived reasonableness of *misrepresentations* or violations of *the standpoint rule*. The relationship between misrepresentation and rhetorical success has then only recently begun to receive empirical attention.

One of the first experimental studies in this respect comes from Bizer et al. (2009), who asked participants to indicate their preference between two fictitious candidates competing for public office based on their statements, one of which either did or did not include a straw man argument. They also manipulated personal relevance by telling participants that the candidate applied for the position either in their state or in a distant one. Their findings indicate that, across the entire sample, the presence or absence of the straw man had no effect on persuasive outcomes. Crucially, however, a significant interaction with the personal relevance variable was detected, such that the inclusion of the straw man positively affected preference for a candidate *in the low-relevance condition* but had no effect under *high personal relevance*. Additionally, they also observed that, holding personal relevance constant, participants high in need for structure (i.e., motivated to adopt an attitude quickly) were more persuaded by the straw man that those low in need for structure. As the authors point out, these results suggest that the straw man has more persuasive appeal when people are not prone to process the message carefully, whether due to low personal relevance or individual disposition (2009, p. 224).

Schumann et al. (2019, 2021) provided further empirical evidence on the straw man’s effectiveness by investigating, among other things, *linguistic factors* that may impact its persuasiveness. In one of their experimental studies, they contrasted misrepresentations of arguments introduced with the French causal connective *puisque* (“since”) with the same distortions without connective and found a significant decrease in acceptability when the straw man was introduced with the connective. This finding, the authors highlight, point to how presentational differences can lead to differences in persuasive impact as they can act as signposts “alerting participants to the possible presence of misattributed content” (2021, p. 365). Additionally, across their studies, the authors found that straw-manning the opposition led to lower agreement with the speaker than comparable non-fallacious messages, providing further evidence on the relative ineffectiveness of the strategy.

Nonetheless, as mentioned, direct evidence on how the straw man fallacy specifically affects reasonableness judgments remains limited. Moreover, no studies to date appear to have investigated the impact of straw-manning on credibility judgments, despite the important role that *ethos* plays in argumentative discourse and persuasive communication (see O’Keefe 2016; Petty et al. 1981; Sperber et al. 2010). Finally, the rhetorical effects of the converse practice of presenting a faithful and clear reformulation of an opponent’s position before attempting to refute it (Dennett 2013; Rapoport 1961) has received limited attention in the literature. The experiments presented in this paper address these research gaps, aiming to clarify the relationship between adhering to normative standards in representing the opposition and rhetorical success.

## The Experimental Study: Introduction and Hypotheses

The experiments presented in this paper experimentally compare the rhetorical effects of messages that contain *faithful reformulations* of an opponent’s position with those that contain *misrepresentations* or *no reformulation* of the opposition. Specifically, evidence is provided on the impact of the three message types on *perceived trustworthiness*, *reasonableness judgments*, and *persuasive outcomes*.

The pre-registered^9^ hypotheses are as follows:

H1a: Misrepresentation will lead to lower trustworthiness ratings than both accurate reformulation and no reformulation.

H1b: Accurate reformulation will lead to higher trustworthiness ratings than no reformulation^10^.

H2: Misrepresentation will lead to lower reasonableness ratings than both accurate reformulation and no reformulation.

H3: Misrepresentation will lead to lower persuasiveness ratings than both accurate reformulation and no reformulation.

The first hypothesis, concerning the effects of the reformulation types on perceived source trustworthiness, specifies all pairwise comparisons. Prediction H1a is grounded on the observation that speakers who misrepresent an opponent’s position commit a violation of dialectical requirements by providing a *nonveridical* account of the opposition. Accordingly, it is reasonable to expect that speakers who engage in misrepresentation will be perceived as less willing to convey an accurate representation of reality, which aligns with how trustworthiness is operationalized in this study and generally understood in the literature (see Sect. 4 and O’Keefe 2016, pp. 292–294). Prediction H1b is based on theoretical observations on the benefits of providing a clear and faithful reformulation of the opposition (see Sect. 2.1; Dennett 2013; Rapoport 1960). The assumption is that providing an accurate reformulation demonstrates a clear and faithful understanding of the opposition and will therefore be perceived as evidence of an arguer’s willingness to offer a veridical account of reality.

The second hypothesis, which pertains to reasonableness judgments, predicts that messages that contain misrepresentations will be perceived as less reasonable than those that contain faithful reformulations and no reformulation. The prediction is based on van Eemeren et al.’s empirical findings that ordinary arguers tend to perceive messages that violate dialectical requirements, i.e., fallacies, as significantly less reasonable than non-fallacious moves (2009, 2012). Since misrepresentation constitutes the only normative violation in the items, the hypothesis concerns only comparisons with the misrepresentation condition.

Finally, the third hypothesis predicts that misrepresentation will lead to lower persuasive outcomes overall. This hypothesis is based on previous findings, which point to the relative ineffectiveness of the strategy, particularly under low personal-relevance conditions and for participants high in need for structure (Bizer et al. 2009; Schumann et al. 2019, 2021).

It should be noted that the hypotheses pertain to the effects of misrepresentation independent of participants’ detection of the distortion as no mediation analyses were performed. While the experimental material was pre-tested for perceived accuracy (see Sect. 4.2), the main experiments did not include measures assessing identification of the distortion^11^. This design decision was made to avoid measurement reactivity and demand effects that could compromise experimental conclusions.

## Methods

Three experiments are presented. The material and conditions are consistent throughout the experiments, with the key distinction being the different response variables assessed. An overview of the study will be provided first, followed by details on the measures used in each experiment^12^.

### Message Stimuli, Design, and Experimental Manipulations

Participants were presented with six scenarios^13^ in which two speakers, Speaker A and Speaker B, engage in an argumentative exchange about two charitable options, Charity A and Charity B. This context was selected based on prior research in experimental psychology showing that, when faced with charitable-giving dilemmas, participants are amenable to changing their attitudes in response to new information (Caviola et al. 2020), making these scenarios well-suited for experimentally studying persuasive effects. An additional benefit of the charity donation context is that it allows for the study of attitude change on consequential issues while avoiding the potential confounds associated with overtly political content.

**Fig. 1:**
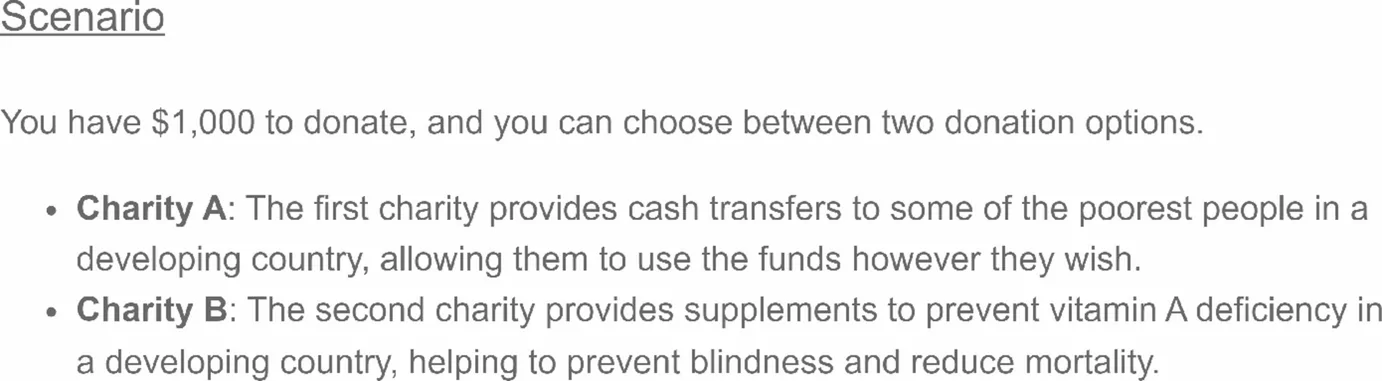
Example of scenario as seen by participants

All the scenarios were built based on previous research in the psychology of effective giving and real-world systematic comparisons of charitable programs (Caviola et al. 2020; MacAskill 2015; Schubert and Caviola 2024). For example, participants were shown dilemmas involving a choice between donating to a charity that helps an identifiable victim versus one that helps non-identifiable victims, or between a non-profit with low overhead costs and moderate cost-effectiveness versus one with high overhead costs but greater cost-effectiveness (see Fig. 1 for an example).

**Fig. 2:**
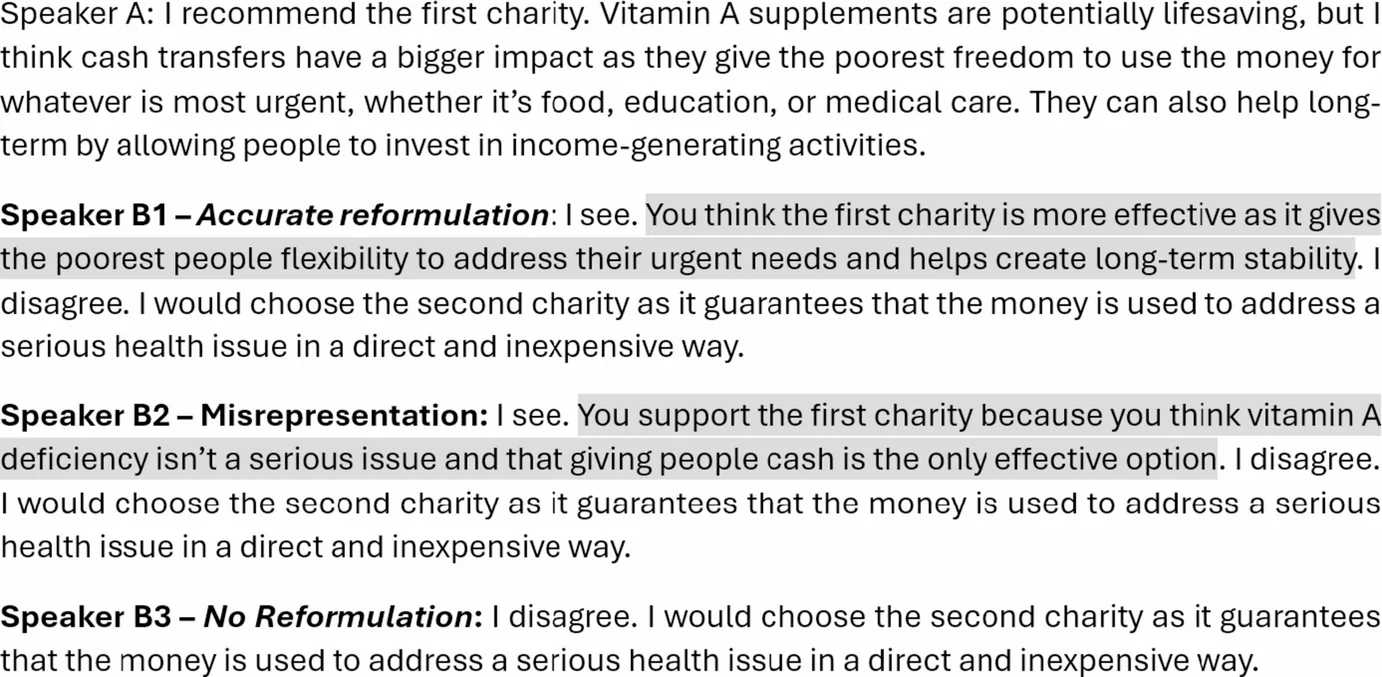
Example of dialogue from a single item. The figure shows all three experimental conditions

After reading the scenario, participants were presented with an argumentative exchange between two speakers. In all cases, Speaker A presents two supporting arguments for Charity A. The experimental manipulation then occurs with Speaker B’s response, who either (i) accurately reformulates, (ii) misrepresents, or (iii) does not reformulate Speaker A’s contribution before providing a supporting argument for Charity B. The scenario, Speaker A’s contribution, and Speaker B’s supporting argument remain identical across conditions, with the only difference being the presence and accuracy of the reformulation (see Fig. 2 for a sample item). The experimental design therefore features three experimental conditions:

1. Accurate reformulation: The speaker provides a faithful reformulation of the opponent’s position, followed by a supporting argument for their own position.
2. Misrepresentation: The speaker provides an inaccurate reformulation of the opponent’s position, followed by the same supporting argument for their own position.
3. No reformulation: The speaker presents only a supporting argument for their own position, without reformulating the opponent’s contribution.

To control for potential confounds related to length, the accurate and inaccurate reformulations are matched in word count. The design is within-subject, counterbalanced using a Latin square design. Participants were randomly assigned to one of three lists, each containing all six items and all conditions, but only one condition per item so that no participant was exposed to the same item in more than one condition. The items were presented in random order to minimize potential order effects.

### Message Pre-testing

A pre-test of the experimental material was conducted to assess perceived accuracy, i.e., whether the misrepresentations in the experimental material were perceived as *inaccurate* and the faithful reformulations as *accurate*. This test therefore serves to check whether the manipulation is likely to have the intended psychological effect.

The decision to test perceived accuracy prior to the main studies was made to avoid the risk of compromising experimental conclusions by incorporating such a measure into the main experiments. Researchers in experimental psychology have noted that manipulation checks embedded within a main study may affect participants’ responses to the dependent variable (e.g., persuasiveness) by influencing how participants process the messages (measurement reactivity) or by inadvertently revealing the researcher’s hypothesis (demand effects) (Fayant et al. 2017; Hauser et al. 2018). These concerns are particularly relevant to experimental work on the effectiveness of the straw man, as previous research suggests that variations in processing can interact with the strategy’s persuasive impact (Bizer et al. 2009). In this respect, researchers have long recommended *pre-testing* manipulations (Aronson and Carlsmith 1968; Fayant et al. 2017; Hauser et al. 2018).

For the pre-test, 81 participants were recruited through the crowdsourcing platform Prolific. The sample was balanced by sex and confined to participants with English as their first language. Table 1 contains information on the participants recruited for the pre-test. As the *no reformulation* condition does not contain a representation of the opposition, the pre-test used a two-list design including only the accurate reformulation and misrepresentation conditions^14^.

**Table 1:** Sample demographics, completion times, and remuneration for pre-test

Characteristic	Value
Sample size	81
Age (years)	
*m (sd)*	41.26 (16.04)
*Range*	[18–83]
Gender	
*Female*	41
*Male*	39
*Other*	1
Completion (min)	
*Mdn (IQR)*	11.65 (8.28)
Remuneration	£ 0.80

Remuneration rates are provided by Prolific based on estimated median completion time. M, sd, mdn, and IQR indicate mean, standard deviation, median, and interquartile range, respectively

Participants read and agreed to a consent form, provided their Prolific ID, and were presented with the six dialogues (see Sect. 4.1). After each dialogue, they were asked “How accurately does Speaker B represent Speaker A’s contribution?”. Responses were recorded on a seven-point scale ranging from “Not at all accurately” (1) to “Very accurately” (7) with “Undecided” (4) at the midpoint of the scale. At the end of the study, participants provided demographic information.

To account for both by-participant and by-item variability, the perceived accuracy data were analyzed using a linear mixed-effects model (LMM) in R (version 4.4.1; R Core Team 2024), implemented with the *lmer* function from the *lme4* package (Bates et al. 2015). The final model included random intercepts for participants and items as well as by-participant random slopes^15^.

**Fig. 3:**
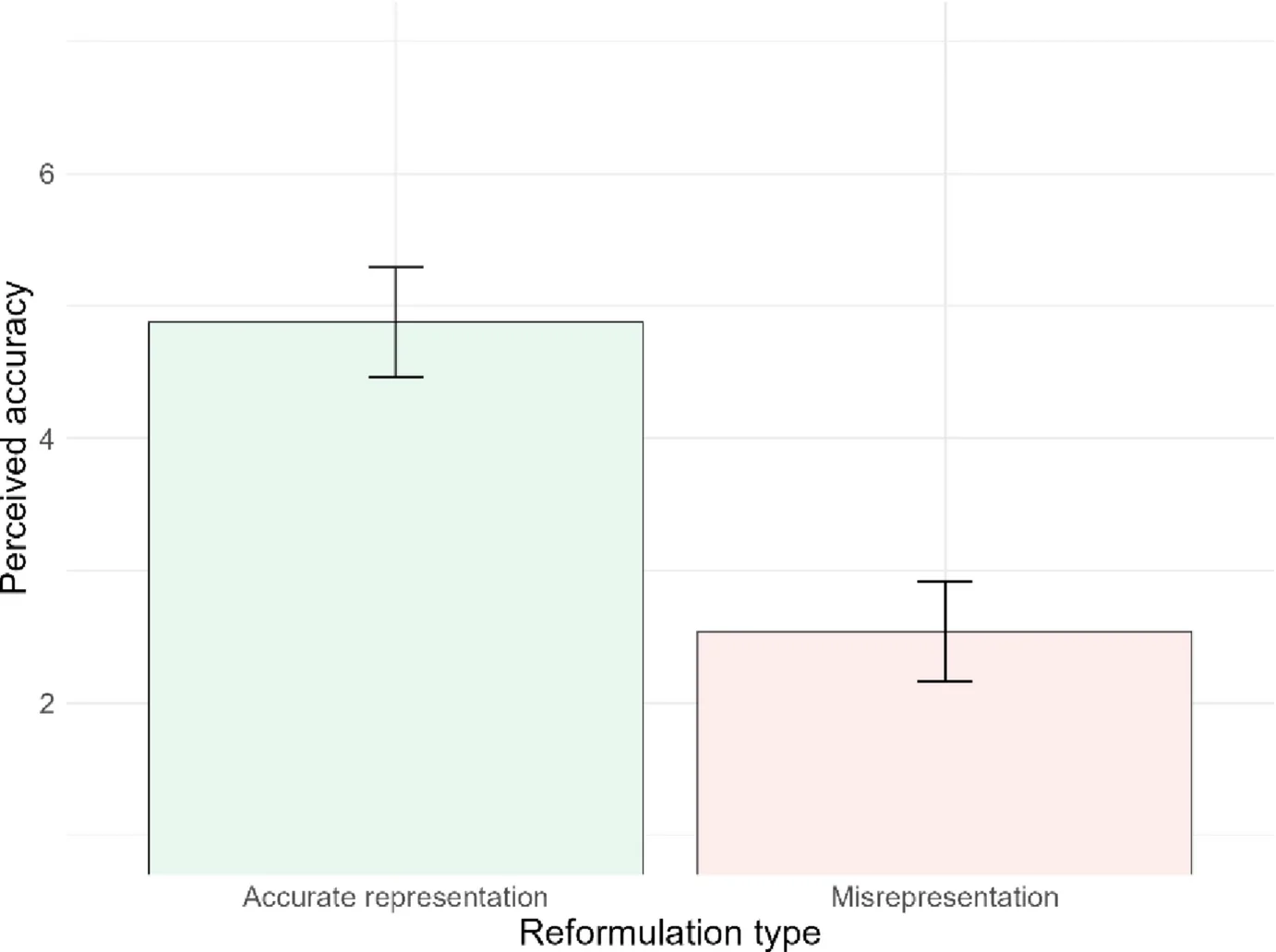
Perceived accuracy by experimental condition (pre-test). Note. Error bars represent 95% confidence intervals

The model’s output showed that the mean perceived accuracy value in the misrepresentation condition was significantly lower (M = 2.54, 95% CI [2.16, 2.91]) than that in the accurate representation condition (M = 4.88, 95% CI [4.46, 5.29]). The results of the LMM are summarized in Table 2. The mean perceived accuracy and confidence intervals by experimental condition are displayed in Fig. 3. Moreover, post hoc intercept-only LMMs indicated that responses in both the misrepresentation and the accurate representation conditions significantly differed from the scale’s midpoint (“undecided”) (*p* <.001), with mean ratings in the former falling significantly below the midpoint and those in the latter significantly above it. 

**Table 2:** Summary of the linear mixed model for the pre-test, comparing accurate reformulation and misrepresentation, with perceived accuracy as the dependent variable, *Reformulation condition* as the fixed effect, *participant* and *item* as a random intercept effect, and *participant* as random slope

Model/fixed effects	Estimate (β)	SE	t-value	*p*(>|t|)	Std. β
*value ~ condition + (1 + condition | Participant) + (1 | item)*					
Intercept (Accurate)	4.88	0.21	23.11	< 0.001***	0.55
Misrepresentation	−2.35	0.24	−9.89	< 0.001***	−1.11

Estimate (β) represents unstandardized regression weights. SE represents standard errors. Standardized β provides the standardized effect size

The results indicate that participants rated faithful representations as *accurate*, significantly more so than distortions, which were perceived as *inaccurate*, supporting the conclusion that the manipulation was perceived as intended.

### Participants and Procedures

For the main experiments, 625 participants in total were recruited. For the analysis, 25 were excluded for failing at two or more comprehension checks, not completing the study, or, in the case of Experiment 2, reporting having studied argumentation theory or pragma-dialectics in formal courses^16^. The final sample consisted of 600 participants in total: 200 recruited independently for each of the three experiments.

The sample size was determined through simulation-based power analysis using linear mixed-effect models in R. The fixed effect size was defined as a 0.20 difference between conditions, namely − 0.20, 0.00, and 0.20 on a 7-point scale. Variance components were set to 0.80 for participants, 0.40 for items, and 2.00 for residuals. The resulting Cohen’s *d* was 0.183. Given an alpha level of 0.05, a sample size of 200 participants yielded an estimated power of 91.20% [95% CI: 86.98, 94.40]^17^. The decision to use conservative expected differences in the power analysis is based on existing meta-analytical evidence, which consistently points to relatively small mean effect sizes connected to persuasive message design choices (see Dillard et al. 1984; O’Keefe and Hoeken 2021; Rains et al. 2018; Weber and Popova 2012). The sample was balanced by sex and confined to speakers of English as a first and fluent language. Table 3 details sample demographics, completion times, and payment for Experiments 1, 2, and 3.

**Table 3:** Sample demographics, completion times, and remuneration for the main experiments

	Experiment 1 Trustworthiness	Experiment 2 Reasonableness	Experiment 3 Persuasiveness
Sample size	200	200	200
Age (years)			
*m (sd)*	38.13 (12.99)	39.25 (13.99)	34.88 (13.11)
*Range*	[19–74]	[18–81]	[18–68]
*Gender*			
Female	98	100	98
Male	102	96	99
Other	0	4	3
Completion (min)			
*mdn (IQR)*	11.76 (9.68)	7.42 (5.68)	11.51 (9.96)
Remuneration	£2.00	£1.00	£1.50

Remuneration rates are provided by Prolific based on estimated median completion time. M, sd, mdn, and IQR indicate mean, standard deviation, median, and interquartile range, respectively

After agreeing to a consent form, participants entered their Prolific ID. In each experiment, they were then randomly assigned to one of three lists (see Sect. 4.1) and read a series of six dialogues. After reading each dialogue, participants provided their ratings on the relevant scales (see below for the scales used in each experiment). At the end of the study, participants provided demographic information, including age, gender identity, and language.

### Measures

#### Experiment 1: Perceived Trustworthiness

The key response variable in the first experiment was perceived speaker trustworthiness. Perceived trustworthiness (sometimes also called character, safety or benevolence) is understood in this context as a dimension of perceived speaker credibility: it is the “the assessment of (roughly) whether the communicator will likely be inclined to tell the truth as he or she sees it” (O’Keefe 2016, p. 293). The term trustworthiness is therefore used here in line with persuasive communication research to refer to only one dimension of credibility^18^. It corresponds to *benevolence* in the epistemic vigilance literature (Sperber et al. 2010) and maps onto the *eunoia*, or *goodwill*, component in the *phronesis*, *eunoia*, *arete* Aristotelian tripartition (Aristotle 1984).

To measure perceived trustworthiness, a six-item 7-point semantic differential scale adapted from Tuppen (1974) and Yalch and Elmore-Yalch (1984) was employed. The six pairs of items used for the perceived trustworthiness scale were the following:

1. Dishonest–Honest.

2. Unfair–Fair.

3. Untrustworthy–Trustworthy.

4. Insincere–Sincere.

5. Closed-minded–Open-minded.

6. Biased–Unbiased.

The scale items were presented in random order to minimize item order effects, as recommended by previous research (McCroskey 1966; Rubin 2004).

#### Experiment 2: Perceived Reasonableness

Experiment 2 has perceived reasonableness as its dependent variable. We adopt the definition of reasonableness articulated in pragma-dialectics, whereby a reasonable argumentative move is one that aligns with dialectical standards for a critical discussion (see Sect. 2; van Eemeren and Grootendorst 2004).

To measure perceived reasonableness, the scale used by van Eemeren et al. (2009, 2012) was replicated. The scale has been repeatedly employed to assess judgments of the reasonableness of fallacious and non-fallacious discussion moves. Experiment 2 takes on board van Eemeren et al.’s recommendation to empirically investigate the relationship between reasonableness and persuasiveness (2012, p. 51). It complements measures from persuasive communication research (Experiment 1 and 3) with a measure from pragma-dialectical effectiveness research (Experiment 2).

After each of the six dialogues between Speaker A and Speaker B, Participants were asked: “How reasonable do you think B’s response was?”. Responses were recorded on a 7-point scale ranging from “very unreasonable” (1) to “very reasonable” (7).

Experiment 2 aimed at examining whether ‘ordinary’ participants’ judgments about the reasonableness of misrepresentational and non-misrepresentational contributions align with a theoretically motivated assessment. For this reason, the pragma-dialectical approach to reasonableness was not specified in the instructions. Following van Eemeren and colleagues, participants were simply asked for judgments concerning “what is or is not permissible in a discussion, what is or is not reasonable” (2009, p. 66)^19^.

#### Experiment 3: Persuasiveness

Experiment 3 measured effects on persuasiveness. Persuasive outcomes were assessed in the experiment at two levels: attitude and behavioral intention (see Hoeken 1994; O’Keefe 2016).

To measure attitude, a three-item, 7-point scale adapted from Allen et al. (1990) was employed. Specifically, after reading each dialogue, participants were asked the following questions regarding both Speaker A’s and Speaker B’s contributions:

1. Do you agree with the speaker?

2. Are the speaker’s arguments persuasive?

3. Did the message persuade you on the issue?

Responses were given on a 7-point scale from “not at all” (1) to “completely” (7). The scale items were presented in random order.

To measure behavioral intention, a 7-point comparative scale was used (see O’Keefe 2016, p. 31). Given that the experimental material presented scenarios resembling those in Caviola et al. (2020), involving a discussion about two charities (Charity A and Charity B) for a $1000 contribution, we used the same scale they employed to measure donation intention. Specifically, participants responded to the question “Of the two donation options, which one would you choose?” using a 7-point scale ranging from “Definitely Charity A” (1) to “Definitely Charity B” (7) with “Unsure” at the midpoint (4).

## Results and Discussion

### Experiment 1: Results and Discussion

To examine the impact of reformulation type on perceived trustworthiness, a linear mixed-effects model (LMM) was fitted in R (version 4.4.1; R Core Team 2024) using the *lmer* function from the *lme4* package (Bates et al. 2015)^20^. Following the best practices suggested by Barr et al. (2013), the LMM included the maximal random effects structure justified by the design. The contribution of random slopes to the model was evaluated by comparing nested models using likelihood ratio tests via the *anova* function. Reformulation type was included as fixed effect, with the *no reformulation* condition as the reference level using treatment coding. The final model specified random intercepts for participants and items as well as by-participant and by-item random slopes. Compared to a random-intercepts-only model, the addition of random slopes significantly improved model fit: Δ*χ2* = 1449.2, Δdf = 10, *p <.*001. Pairwise comparisons between levels were performed with Tukey’s adjustment for multiple comparisons.

**Fig. 4:**
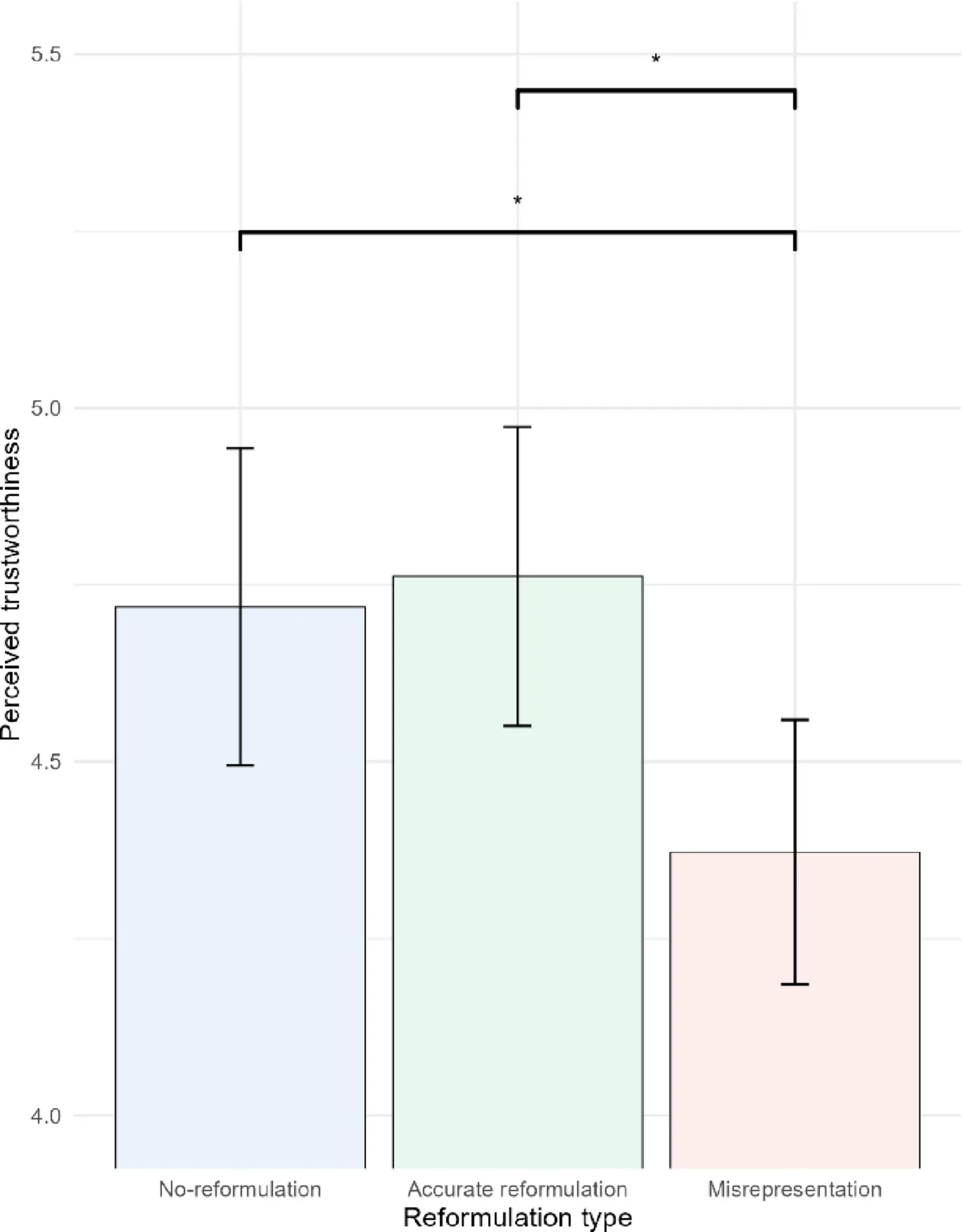
Perceived trustworthiness by experimental condition (Experiment 1). Note. Error bars represent 95% confidence intervals. *indicates p < .05

The results show that participants rated the speaker (Speaker B) as significantly more trustworthy in the *accurate reformulation* condition (*estimate* = 0.39, *SE* = 0.13, *p* <.05) and in the *no reformulation* condition (*estimate* = 0.35, *SE* = 0.13, *p* <.05), compared to the *misrepresentation* condition. However, no significant difference was found between *accurate reformulation* and *no reformulation* (*p* >.05). The mean perceived trustworthiness and confidence intervals by experimental condition are presented in Fig. 4.

These results support H1a, which predicted that *misrepresentation* would lead to lower trustworthiness ratings relative to the other conditions. However, H1b, which predicted an advantage of *accurate reformulation* over *no reformulation*, was not supported. The results of the linear mixed model are shown in Table 4. Pairwise comparisons between conditions are presented in Table 5. An internal consistency analysis indicated that the six-item semantic differential scale adapted from Tuppen (1974) and Yalch and Elmore-Yalch (1984) demonstrated excellent internal reliability (Cronbach α = 0.93).

**Table 4:** Summary of the linear mixed model for experiment 1, comparing *accurate reformulation*, *misrepresentation*, and *no reformulation*, with perceived trustworthiness as the dependent variable

Model/Fixed effects	Estimate (β)	SE	t-value	*p*(>|t|)
*value ~ Condition + (Condition | item) + (Condition | Participant)*				
Intercept (No reformulation)	4.72	0.11	41.27	< 0.001***
Accurate representation	0.04	0.21	0.40	0.915
Misrepresentation	− 0.35	0.12	−2.73	0.006**

Estimate (β) represents unstandardized regression weights. SE represents standard errors. The table also reports *t*-values and corresponding *p*-values

**Table 5:** Pairwise comparisons of trustworthiness ratings by reformulation condition

Contrasts	estimate	SE	*p*-value	Cohen’s d
Accurate reformulation–Misrepresentation	0.39	0.13	0.027*	−0.34
No reformulation–Misrepresentation	0.35	0.13	0.036*	−0.30
No Reformulation –Accurate reformulation	− 0.04	0.11	0.916	−0.04

The table includes estimated mean differences (estimate), standard errors (SE), p-values, and Cohen’s *d* effect sizes

### Experiment 2: Results and Discussion

To analyze the effects of reformulation type on reasonableness judgments, a linear mixed model was fitted in R using the same function and procedure as in Experiment 1^21^. The final model included random intercepts for participants and items as well as a by-participant random slope. This represented the most complex design consistent with the experiment that resulted in a non-singular fit and significantly improved the model. Compared to a random-intercepts-only model, the addition of the by-participant random slope significantly improved model fit: Δ*χ2* = 69.87, Δdf = 5, *p <.*001. As in the previous experiment, pairwise comparisons were performed with Tukey’s adjustment.

**Fig. 5:**
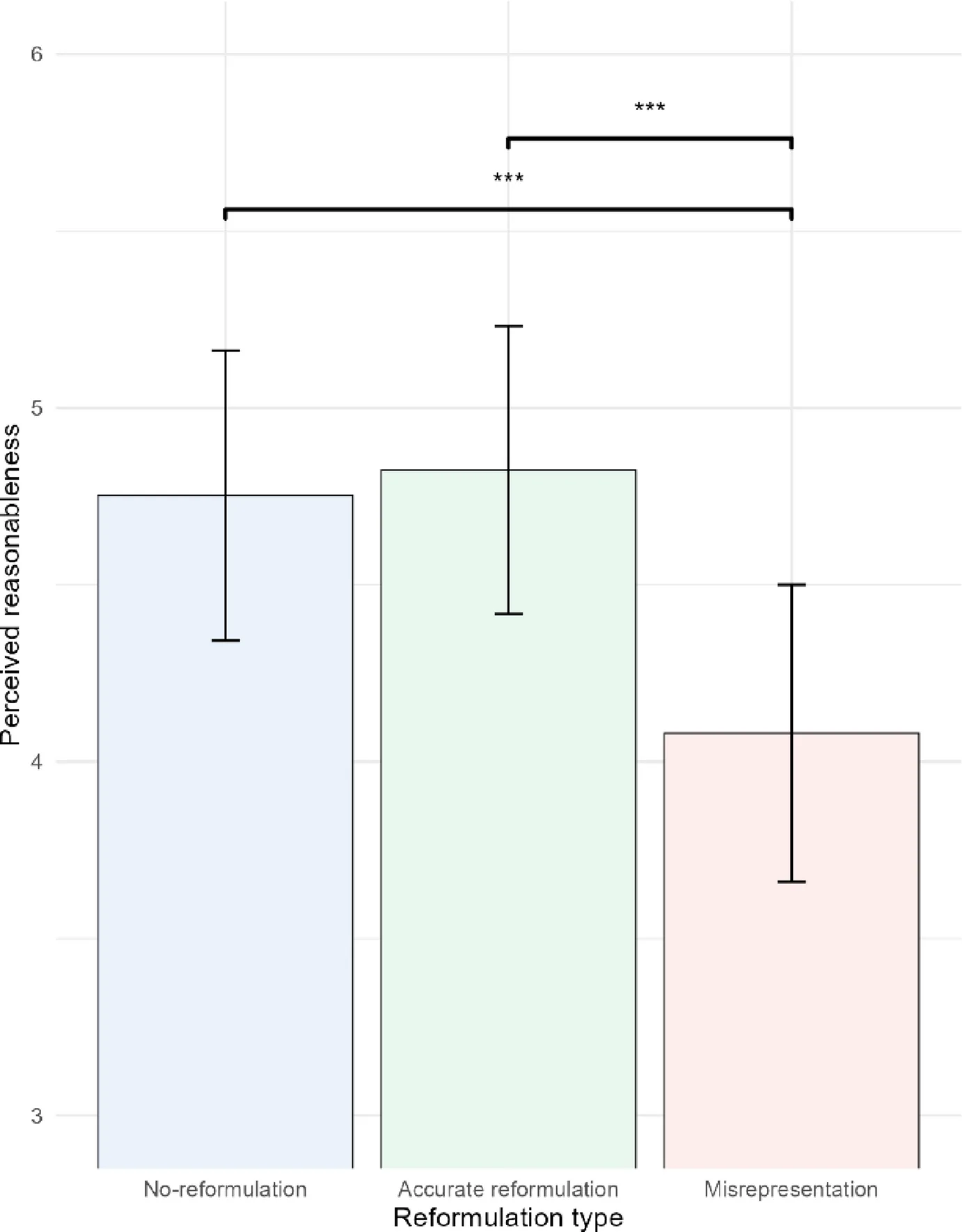
Perceived reasonableness by experimental condition (Experiment 2). Note. Error bars represent 95% confidence intervals. *** indicates p < .001

The results show that participants rated the message in the *misrepresentation* condition as significantly less reasonable than both in the *accurate reformulation* condition (*estimate* = −0.74, *SE* = 0.14, *p* <.001) and in the *no reformulation* condition (*estimate* = −0.67, *SE* = 0.14, *p* <.001). Figure 5 shows the mean perceived reasonableness and confidence intervals by experimental condition.

These results are consistent with H2, supporting the prediction that *misrepresentation* would result in lower reasonableness judgments compared to the other conditions. Table 6 shows the results of the linear mixed model, and pairwise comparisons between conditions are presented in Table 7.

**Table 6:** Summary of the linear mixed model for experiment 2, comparing *accurate reformulation*, *misrepresentation*, and *no reformulation*, with perceived reasonableness as the dependent variable

Model/Fixed effects	Estimate (β)	SE	t-value	*p*(>|t|)
*value ~ Condition + (1 + Condition | Participant) + (1 | item)*				
Intercept (No reformulation)	4.75	0.21	22.75	< 0.001***
Accurate representation	0.07	0.11	0.67	0.504
Misrepresentation	− 0.67	0.14	−4.70	< 0.001***

Estimate (β) represents unstandardized regression weights. SE represents standard errors. The table also reports *t*-values and corresponding *p*-values

**Table 7:** Pairwise comparisons of reasonableness ratings by reformulation condition

Contrasts	estimate	SE	t-ratio	*p*-value	Cohen’s d
Accurate reformulation–Misrepresentation	0.74	0.14	5.29	< 0.001***	0.55
No reformulation–Misrepresentation	0.67	0.14	4.70	< 0.001***	0.49
No reformulation –Accurate representation	− 0.07	0.14	−0.67	0.782	−0.05

The table includes estimated mean differences (estimate), standard errors (SE), t-ratios, p-values, and Cohen’s *d* effect sizes

### Experiment 3: Results and Discussion

To test the hypothesis regarding persuasive outcomes (H3), attitude and behavioral intention were analyzed separately, in line with their conceptual differences. Accordingly, two linear mixed models with a maximal random effects structure were fitted in R, using the same function and package as in the previous experiments^22^.

In the case of the attitude data, the final model included random intercepts for participants and items as well as by-participants random slope. The addition of the by-participant random slope improved the model compared to a model with random intercepts only: Δ*χ2* = 471.34, Δdf = 5, *p <.*001. For the behavioral intention data, the final model included only random intercepts for participants and items. Adding random slopes did not significantly improve model fit and led to a singular fit. The random-intercepts-only model was therefore selected.

**Fig. 6:**
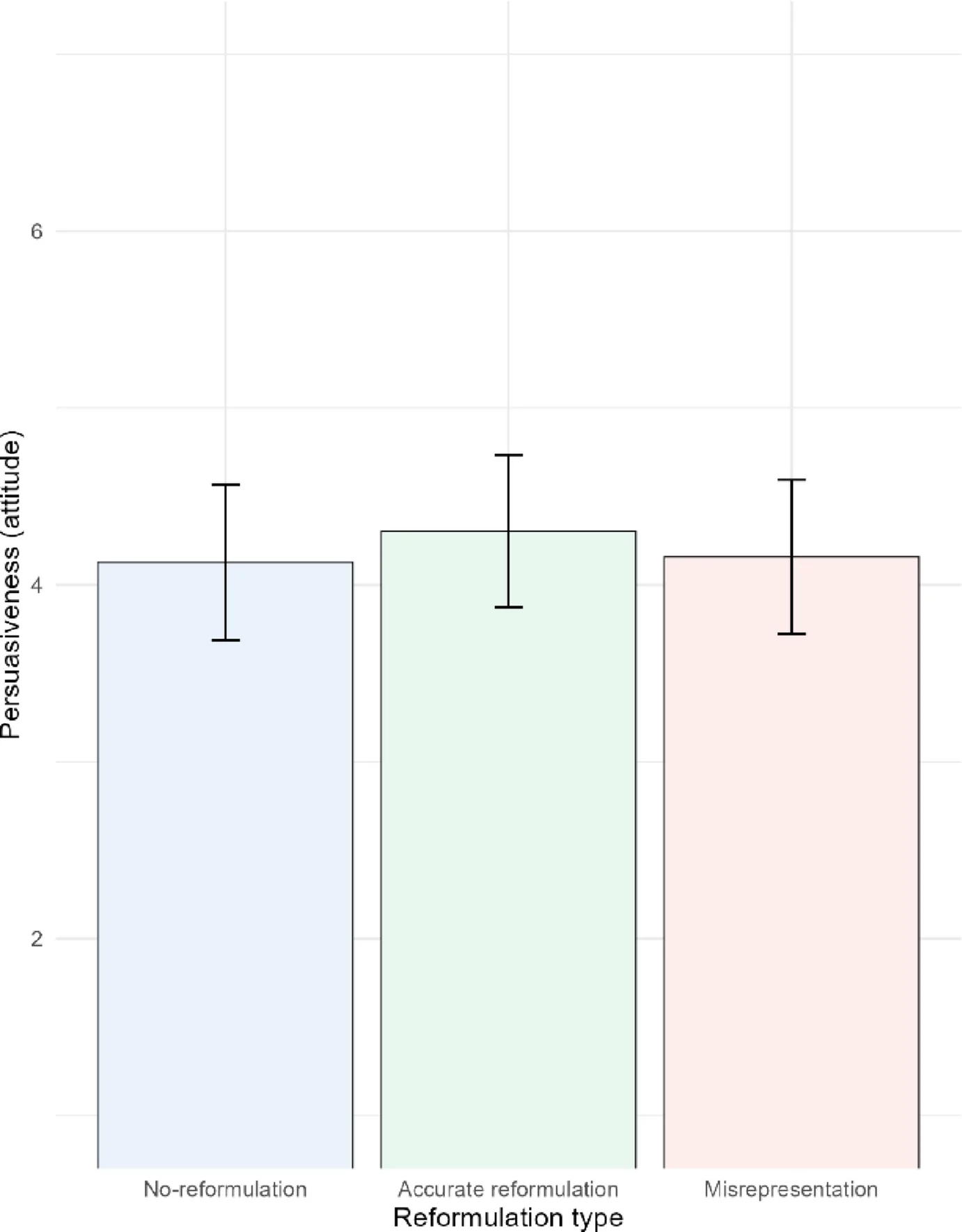
Persuasiveness score (attitude variable) by experimental condition (Experiment 3). Note. Error bars represent 95% confidence intervals

The results for the attitude-level persuasiveness data do not reveal significant differences between conditions. The mean persuasiveness scores for the attitude variable, along with confidence intervals by experimental condition, are presented in Fig. 6.

Table 8 provides the results of the relevant linear mixed model, and pairwise comparisons between conditions are shown in Table 9. An internal consistency analysis indicated that the three-item scale adapted from Allen et al. (1990) demonstrated excellent internal reliability (Cronbach α = 0.93).

**Table 8:** Summary of the first linear mixed model for experiment 3, comparing *accurate reformulation*, *misrepresentation*, and *no reformulation*, with attitude-level persuasiveness as the dependent variable

Model/Fixed effects	Estimate (β)	SE	t-value	*p*(>|t|)
*value ~ Condition + (1 + Condition | Participant) + (1 | item)*				
Intercept (No reformulation)	4.12	0.22	18.39	< 0.001***
Accurate representation	0.17	0.11	1.66	0.098
Misrepresentation	0.03	0.12	0.25	0.801

Estimate (β) represents unstandardized regression weights. SE represents standard errors. The table also reports *t*-values and corresponding *p*-values

**Table 9:** Pairwise comparisons of attitude-level persuasiveness ratings by reformulation condition

Contrasts	estimate	SE		*p*-value	Cohen’s d
Accurate reformulation–Misrepresentation	0.15	0.10	0.306		0.11
No reformulation–Misrepresentation	−0.03	0.12	0.966		−0.02
No Reformulation –Accurate representation	− 0.17	0.11	0.225		−0.13

The table includes estimated mean differences (estimate), standard errors (SE), z-ratios, p-values, and Cohen’s *d* effect sizes

**Fig. 7:**
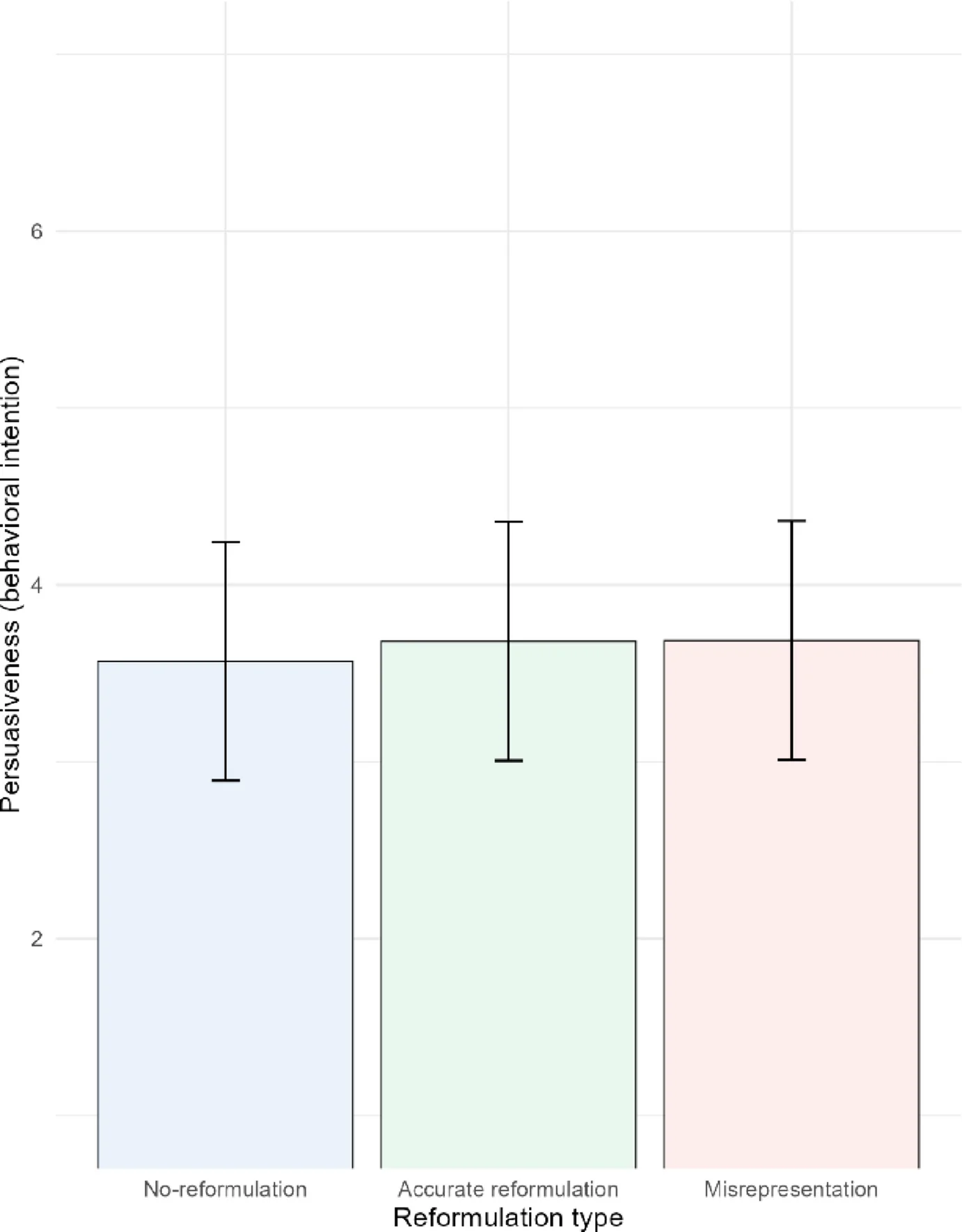
Persuasiveness score (behavioral intention variable) by experimental condition (Experiment 3). Note. Error bars represent 95% confidence intervals

Similarly, the analysis of persuasiveness at the behavioral intention level showed no significant differences between conditions. Figure 7 presents the mean scores for the behavioral intention variable as well as 95% confidence intervals. The results of the second linear mixed model are shown in Table 10, and Table 10 presents pairwise comparisons between conditions.

**Table 10:** Summary of the first linear mixed model for Experiment 3, comparing *accurate reformulation*, *misrepresentation*, and *no reformulation*, with intention-level persuasiveness as the dependent variable.

Model/Fixed effects	Estimate (β)	SE	t-value	*p*(>|t|)
*value ~ Condition + (1 + Condition | Participant) + (1 | item)*				
Intercept (No reformulation)	3.57	0.34	10.36	< 0.001***
Accurate representation	0.11	0.14	0.80	0.427
Misrepresentation	0.12	0.14	0.83	0.407

Estimate (β) represents unstandardized regression weights. SE represents standard errors. The table also reports *t*-values and corresponding *p*-values

**Table 11:** Pairwise comparisons of intention-level persuasiveness ratings by reformulation condition

Contrasts	estimate	SE	*p*-value	Cohen’s d
Accurate reformulation–Misrepresentation	−0.00	0.14	0.706	0.00
No reformulation–Misrepresentation	−0.11	0.14	0.685	−0.08
No Reformulation –Accurate representation	− 0.11	0.14	0.999	−0.08

The table includes estimated mean differences (estimate), standard errors (SE), p-values, and Cohen’s *d* effect sizes

These results do not support H3, which predicted that misrepresentation would lead to lower persuasiveness when compared to the other conditions.

## General Discussion and Conclusions

The results from Experiment 1, 2, and 3 support hypotheses 1a and 2, but do not support hypotheses 1b and 3.

Turning first to the findings on misrepresentation, the present study provides, as far as we are aware for the first time, experimental evidence on how distorting an opponent’s contribution affects speaker *ethos* or perceived credibility. Specifically, in line with the prediction, the results from Experiment 1 suggest that misrepresenting the opposition negatively impacts trustworthiness judgments compared to messages with accurate reformulation or no reformulation. This finding contributes to research on the rhetorical effects of the straw man (Bizer et al. 2009; Schumann 2022) by highlighting a rhetorical cost associated with the practice.

Relatedly, in Experiment 2, discussion contributions that misrepresented the opposition were perceived as significantly less reasonable than those that did not. This finding adds to the body of work investigating reasonableness judgments in argumentative discourse, particularly within the context of pragma-dialectical effectiveness research (van Eemeren et al. 2009; Eemeren et al. 2012). The results build on previous empirical studies on how ordinary arguers evaluate fallacious moves by showing that lay evaluations of argumentative quality are sensitive to violations of normative standards regarding the representation of the opposition. In this respect, these findings highlight the value of complementing, as advocated by van Eemeren et al. (2012), persuasive communication research with theoretically motivated effectiveness research to investigate the connection between reasonableness and rhetorical effectiveness.

Experiment 3, which investigated persuasive outcomes, further showed that misrepresentation did not offer a significant rhetorical advantage. However, contrary to expectations, distorting the opposition did not result in lower persuasiveness compared to accurate reformulation or no reformulation: no significant effects were observed. While this does not support the initial prediction, it aligns with prior findings from Bizer et al. (2009), who found that the inclusion of a straw man had no effect on persuasive outcomes across their entire samples in two experiments.

The results on persuasiveness may in part be explained by the fact that the supporting argumentation in the present study remained identical across conditions. While participants were sensitive to the accuracy with which a speaker represented the opposition when evaluating reasonableness and trustworthiness, it is plausible that, when asked to report their behavioral intentions and attitudes, their responses were based primarily on whether the reasons provided—as opposed to how accurately the opposition was represented—changed their minds. In this respect, these results emphasize the importance of assessing different outcome variables: the absence of an effect on persuasive outcomes should not be taken to imply a lack of rhetorically relevant consequences for perceived credibility or reasonableness. Indeed, one can separate the quality of the monological, supporting argumentation from the quality of the speaker’s behavior in dialogue. Based on the results, participants seem to, quite reasonably, be able to make this distinction and separate how fairly speakers engage in dialogue from the strength of the position itself.

Taken together, the results indicate that, in the cases investigated, adhering to normative standards (i.e., avoiding misrepresentation) did not reduce rhetorical effectiveness. On the contrary, speakers who misrepresented the opposition paid a price in terms of perceived trustworthiness, and lay participants were sensitive to such violations when evaluating reasonableness. These findings build on O’Keefe’s (2003, 2006) work by providing further evidence that reasonableness and rhetorical effectiveness are not in fundamental conflict, helping to dispel the concern that fallacious contributions are generally more effective. It is important to note that this should not be taken to imply that misrepresentative or unreasonable argumentative practices are *invariably* less persuasive than those that stay within the bounds of dialectical requirements. However, the empirical evidence available thus far does show reasonable argumentation to be generally consistent with, if not advantageous for, rhetorical success, at least for the specific practices that have been experimentally examined.

Turning to the findings on accurate reformulation, while the results show this practice to be more rhetorically effective than misrepresentation, they do not suggest an advantage over no reformulation, contrary to expectations. This may partly be explained by the fact that the messages reformulated the opponent’s arguments without attempting to directly refute them. Using terminology from persuasive communication research, the messages in the accurate reformulation condition can be classified as *nonrefutational* two-sided messages (Jackson and Allen 1987; O’Keefe 2016). Unlike one-sided contributions in the *no reformulation* condition, these messages acknowledged opposing arguments, but they did not contain a rebuttal aimed at showing the represented position to be defective. In this respect, the results are consistent with previous research, which found that nonrefutational twosided messages on nonadvertising issues do not differ in credibility and persuasiveness from one-sided messages (O’Keefe 1999).

Previous research on sidedness effects, however, found that *refutational* two-sided contributions resulted in significantly increased credibility and persuasiveness compared to one-sided messages. It would therefore be valuable to explore whether the practice of accurate reformulation recommended by Dennett (2013) and Rapoport (1960, 1961) would be rhetorically advantageous when the opposing views are not only acknowledged but also directly refuted. A future line of inquiry would therefore be to test whether the effects on refutational two-sided messages observed in persuasive communication research extend to dialogical, argumentative exchanges such as those explored in this study.

Furthermore, participants in the experiments were third-person witnesses of argumentative exchanges *between other speakers*, similarly to previous studies on the effectiveness of the straw man (see Aikin and Casey 2022, pp. 197–199). The rhetorical benefits of faithful reformulations theorized by Dennett (2013) and Rapoport (1960, 1961), however, pertain mostly to *second-personal cases*. Specifically, the authors contend that one of the effects of re-expressing an opponent’s position fairly and clearly is to convince *the opponent* that they have been understood, which in turn makes it more likely that *they* will be persuaded (Dennett 2013, p. 33; Rapoport 1961, p. 216). This points to the need for further research examining the second-person impact of faithful reformulation, i.e., its effects on participants who are themselves the target of the representation.

Future studies could then benefit from more closely exploring whether the *detection* of misrepresentation plays a role in how distortion affects persuasion. Previous research indicates that inclusion of a straw man can be rhetorically effective when participants are not prone to carefully process the message, which in turn suggests that the effectiveness of misrepresentation may be mediated by identification of the distortion (Bizer et al. 2009). While the results of the pre-test and main experiments offer some indication of whether the misrepresentations were perceived as such by participants in this study, the absence of a detection measure in the main experiments meant it was not possible to test mediation effects. Incorporating such a measure could help determine whether misrepresentation positively impacts perceived credibility and persuasion when it goes unnoticed. However, it is important to consider the methodological challenges involved in embedding such a measure in the main experiments, as this may lead to demand effects or other participant reactions that affect responses to the outcome variable^23^ (Fayant et al. 2017; Hauser et al. 2018).

It should also be noted that, while the experiments did not show misrepresentation to have an immediate effect on persuasive outcomes, this should not be taken to mean that distorting the opposition may not have *delayed* effects that are outside the scope of this study^24^. As researchers concerned with the pragmatics of the straw man have pointed out (e.g., de Saussure 2018), the decision to distort an opponent’s position may, for instance, affect the unfolding of dialogue by putting the target in a defensive position, leading them to justify their contribution rather than provide supporting argumentation for their standpoint. Furthermore, this study has shown that speakers who distort the opposition pay a price in terms of perceived trustworthiness. This loss of credibility may, in turn, have delayed negative effects on persuasive outcomes, especially for subsequent arguments that rely on the credibility of the speaker. It would therefore be interesting to experimentally explore rhetorically relevant consequences of misrepresentation that go beyond the scope of the exchange shown to participants in this study.

Another potential direction for future research is to investigate whether the findings from this study generalize to persuasive messages on different topics. The experimental material was confined to discussion contributions in the charitable giving context, and it would be valuable to learn whether the effects observed in the present research extend to argumentative exchanges on more polarizing topics that are more likely to involve ideologically motivated reasoning.

## Data Availability

The experimental material, data, and R scripts used in this study are open access and can be found in the Open Science Framework repository at: https://osf.io/ck83z/overview?view_only=2bda0938a4234a6588ed40b5c47de34a .

## Footnotes

1 While the rule, taken literally, pertains to the distortion of standpoints, it can, as others have aptly pointed out, equally be applied to the representation of arguments, which in pragma-dialectics can become sub-standpoints supported by sub-arguments (see Lewiński 2011; Schumann et al. 2019).

2 The straw man can, in turn, take different forms. Aikin and Casey identify and discuss three broad types: (i) the *classic* or *representational form*, distorting the standpoints or arguments put forward by a speaker, (ii) the *selectional form* or *weak man*, selecting the weakest arguments or standpoints from those advanced by an opponent, and (iii) the *hollow man*, attributing completely fabricated views to an opponent (Aikin and Casey 2011).

3 Following Katriel and Dascal’s definition (1989), as adapted by Oswald (2016), commitment is understood here as “content that the speaker can be said to have taken for granted in making his or her utterance and which they cannot retract without causing semantic or pragmatic inconsistencies” (2016, pp. 17–18).

4 In this respect, Oswald (2016), for instance, argues that the cognitive pragmatic account offered by relevance theory (Sperber and Wilson 1995) provides solid methodological tools for the plausible reconstruction of arguments.

5 Dennett calls these principles *Rapoport’s rules* but clarifies that he is presenting his own formulation based on memory of correspondence with Rapoport (2013, p. 34).

6 A detailed discussion of the practice of faithful reformulation and the notion of *steel man* is currently in preparation.

7 As O’Keefe notes, this question is part of a recurring concern regarding the relationship between *normative* and *descriptive* approaches to argumentation (2003).

8 For historical background on the view that dialectic and rhetoric are incompatible paradigms, see, e.g., van Eemeren and Houtlosser 1999; who trace the origins of what they describe as an “infertile ideological division”.

9 Pre-registrations for each experiment can be accessed via the following OSF links: Experiment 1: https://osf.io/z8f2c/?view_only=dfd03db2c02b439ba7917ffc7f3ad323. Experiment 2: https://osf.io/fjbdr/?view_only=7f231ec568f24e1ab7fcbc00a2817623. Experiment 3: https://osf.io/cf6sn/?view_only=78373f077dfe4a8e9246dc40cb9647e3.

10 Note that the comparison between accurate reformulation and inaccurate reformulation is already addressed in H1a.

11 For a more detailed discussion of this design choice, see Sect. 4.2 and 6.

12 The experimental material, data, and R scripts used in this study are open access and can be found in the Open Science Framework repository (Younis 2026) https://osf.io/ck83z/?view_only=b6f1e1ce4897410ba9e5e08fb4bac545.

13 A multiple-message design was employed to avoid issues of ambiguous causal attribution and nonuniform effects across messages (see O’Keefe 2016).

14 As in the main experiments, this is a within-subject design, with the conditions counterbalanced using a Latin square. Each participant was therefore exposed to all conditions but only one condition per item.

15 This model represented the most complex design consistent with the experiment that resulted in a non-singular fit and significantly improved the model. For more details on the statistical analysis procedure followed in this study, see Sect. 5.

16 Given that Experiment 2 seeks to assess, following van Eemeren et al. (2009, 2012), perceived reasonableness in “ordinary arguers” (see Sect. 4.4.2), it was important to ensure participants had not studied the pragma-dialectical conception of reasonableness.

17 The script with the complete procedure for this power analysis is available in the OSF depository (Younis 2026).

18 There is a wealth of factor-analytic research that groups intercorrelated scales measuring perceived credibility (e.g., Applbaum and Anatol 1972; Baudhuin and Davis 1972). The dimension of trustworthiness, as defined here, is one of two dimensions that commonly emerges from these studies, alongside competence.

19 For a detailed discussion of the methodological considerations behind the measure, see van Eemeren et al. 2009.

20 Given that trustworthiness was measured on a 7-point ordinal scale, the data were also analyzed using a Cumulative Link Mixed Model (CLMMs). I wish to thank an anonymous reviewer for this suggestion. The model for this analysis was implemented in R with the CLMM function from the ordinal package (Christensen 2023) and included random intercepts for participants and items. The supplementary analysis confirmed the experimental conclusions observed with the LMM and discussed in the paper. The script and results for this analysis are available in the OSF repository (Younis 2026).

21 As with Experiment 1, the data were also analyzed using a supplementary CLMM. The model was implemented using the same package and included the same random effects as in Experiment 1, and its output confirmed the experimental conclusions. For more details, see the scripts and results on OSF (Younis 2026).

22 As in Experiments 1 and 2, supplementary analyses were conducted using CLMMs following the same procedure and including the same random effects described in Sect. 5.1 and 5.2. The models’ outputs confirmed the experimental conclusions discussed here. The scripts and results of the supplementary analyses are available in the OSF repository (Younis 2026).

23 See the rationale in Sect. 4.2.

24 I wish to thank an anonymous reviewer for their considerations on this point.

## References

[CR1] Aikin, S., and J. Casey. 2011. Straw men, weak men, and hollow men. *Argumentation* 25: 87–105. 10.1007/s10503-010-9199-y.

[CR2] Aikin, S., and J. Casey. 2013. Don’t feed the trolls: Straw men and iron men. *Virtues of Argumentation. Proceedings of the 10th International Conference of the Ontario Society for the Study of Argumentation*. OSSA 10. https://scholar.uwindsor.ca/cgi/viewcontent.cgi?article=1975&context=ossaarchive

[CR3] Aikin, S., and J. Casey. 2016. Straw men, iron men, and argumentative virtue. *Topoi* 35: 431–440. 10.1007/s11245-015-9308-5.

[CR4] Aikin, S., and J. Casey. 2022. *Straw Man Arguments*. Bloomsbury.

[CR5] Allen, M., J. Hale, P. Mongeau, S. Berkowitz-Stafford, S. Stafford, W. Shanahan, P. Agee, K. Dillon, R. Jackson, and C. Ray. 1990. Testing a model of message sidedness: Three replications. *Communication Monographs* 57(4):275–291. 10.1080/03637759009376203

[CR6] Applbaum, R.F., and K.W.E. Anatol. 1972. The factor structure of source credibility as a function of the speaking situation. *Speech Monographs* 39 (3): 216–222. 10.1080/03637757209375760.

[CR7] Aristotle. 1984. *Complete works of aristotle: The revised Oxford translation*, ed. J. Barnes. Princeton University Press. 10.1515/9781400835843

[CR8] Aronson, E., and J.M. Carlsmith. 1968. Experimentation in social psychology. In *The Handbook of Social Psychology*, 2nd ed., ed. L. Gardner, 1–79. Addison-Wesley.

[CR10] Barr, D.J., R. Levy, C. Scheepers, and H.J. Tily. 2013. Random effects structure for confirmatory hypothesis testing: Keep it maximal. *Journal of Memory and Language* 68 (3): 255–278. 10.1016/j.jml.2012.11.001. PMC3881361 24403724

[CR11] Bates, D., M. Mächler, B. Bolker, and S. Walker. 2015. Fitting linear Mixed-Effects models using lme4. *Journal of Statistical Software* 67(1). 10.18637/jss.v067.i01

[CR12] Baudhuin, E.S., and M.K. Davis. 1972. Scales for the measurement of *ethos*: Another attempt. *Speech Monographs* 39 (4): 296–301. 10.1080/03637757209375769.

[CR13] Bizer, G.Y., S.M. Kozak, and L.A. Holterman. 2009. The persuasiveness of the straw man rhetorical technique. *Social Influence* 4 (3): 216–230. 10.1080/15534510802598152.

[CR14] Caviola, L., S. Schubert, and J. Nemirow. 2020. The many obstacles to effective giving. *Judgment and Decision Making* 15 (2): 159–172. 10.1017/S1930297500007312.

[CR15] Christensen, R. H. B. 2023. *ordinal: Regression Models for Ordinal Data* (Version 2023.12–4.1, p. 2023.12–4.1) [Dataset]. 10.32614/CRAN.package.ordinal

[CR19] Dennett, D.C. 2013. *Intuition pumps and other tools for thinking*. W.W. Norton & Company.

[CR17] De Rijk, F. 2024. Principle of charity. In *Migration: A philosophical toolkit*, ed. M. Pauly. University of Groningen. 10.21827/660d42361e623

[CR18] de Saussure, L. 2018. The Straw Man Fallacy as a Prestige-Gaining Device. In *Argumentation and Language—Linguistic, Cognitive and Discursive Explorations*, vol. 32, ed. S. Oswald, T. Herman, and J. Jacquin, 171–190. Springer International Publishing. 10.1007/978-3-319-73972-4_8.

[CR21] Fayant, M.-P., H. Sigall, A. Lemonnier, E. Retsin, and T. Alexopoulos. 2017. On the limitations of manipulation checks: An obstacle toward cumulative science. *International Review of Social Psychology* 30(1):125–130. 10.5334/irsp.102

[CR22] Govier, T. 1981. Uncharitable thoughts about charity. *Informal Logic* 4(1). 10.22329/il.v4i1.2761

[CR23] Hauser, D.J., P.C. Ellsworth, and R. Gonzalez. 2018. Are manipulation checks necessary? *Frontiers in Psychology*. 10.3389/fpsyg.2018.00998. PMC6022204 29977213

[CR24] Hoeken, H. 1994. Evaluating Persuasive Texts: The Problems of How and What to Measure. In *Functional Communication Quality*, ed. L. Van Waes, E. Woudstra, and P. den Van Hoven, 76–87. Brill. 10.1163/9789004484528.

[CR25] Jackson, S. 1992. Virtual standpoints and the pragmatics of conversational argument. In F. H. van Eemeren & J. A. Blair (Eds.), *Argumentation illuminated*. SicSat.

[CR26] Jackson, S., and M. Allen. 1987. Meta-analysis of the effectiveness of one-sided and two-sided argumentation. *Paper Presented at the International Communication Association Convention, Montreal, Canada.*

[CR20] Dillard, J. P., J. E. Hunter, and M. Burgoon. 1984. Sequential-request persuasive strategies. Meta-analysis of foot-in-the-door and door-in-the-face. *Human Communication Research* 10(4):461–488. 10.1111/j.1468-2958.1984.tb00028.x.

[CR27] Katriel, T., and M. Dascal. 1989. Speaker’s commitment and involvement in discourse. In *From sign to text: A semiotic view of communication*, ed. Y. Tôbîn. John Benjamins Publishing Company. 10.1075/fos.20

[CR28] Lewiński, M. 2011. Towards a critique-friendly approach to the straw man fallacy evaluation. *Argumentation* 25: 469–497. 10.1007/s10503-011-9227-6.

[CR29] Lewiński, M., and S. Oswald. 2013. When and how do we deal with straw men? A normative and cognitive pragmatic account. *Journal of Pragmatics* 59:164–177. 10.1016/j.pragma.2013.05.001

[CR30] MacAskill, W. 2015. *Doing Good Better: How Effective Altruism Can Help You Make a Difference*. Penguin Publishing Group.

[CR31] McCroskey, J.C. 1966. Scales for the measurement of ethos. *Speech Monographs* 33 (1): 65–72. 10.1080/03637756609375482.

[CR32] O’Keefe, D.J. 1999. How to handle opposing arguments in persuasive messages: A meta-analytic review of the effects of one-sided and two-sided messages. *Annals of the International Communication Association* 22 (1): 209–249. 10.1080/23808985.1999.11678963.

[CR33] O’Keefe, D. J. 2003. The Potential Conflict Between Normatively-Good Argumentative Practice and Persuasive Success: Evidence From Persuasion Effects Research. In F. H. van Eemeren, J. A. Blair, C. A. Willard, & A. F. Snoeck Henkemans (Eds.), *Anyone Who Has a View: Theoretical Contributions to the Study of Argumentation* (1st ed. 2003, pp. 309–318). Imprint: Springer. 10.1007/978-94-007-1078-8

[CR34] O’Keefe, D.J. 2006. Pragma-dialectics and persuasion effects research. In* Considering Pragma-Dialectics: A Festschrift for Frans H. Van Eemeren on the Occasion of his 60th Birthday*, ed. P. Houtlosser & A. van Rees, 235–243. L. Erlbaum.

[CR35] O’Keefe, D.J. 2016. *Persuasion: Theory and research*. 3rd ed. SAGE Publications.

[CR36] O’Keefe, D.J., and H. Hoeken. 2021. Message design choices don’t make much difference to persuasiveness and can’t be counted on—not even when moderating conditions are specified. *Frontiers in Psychology* 12: 664160. 10.3389/fpsyg.2021.664160. 34267703 PMC8275937

[CR37] Oswald, S. 2016. Commitment attribution and the reconstruction of arguments. In *The psychology of argument: Cognitive approaches to argumentation and persuasion*, ed. F. Paglieri, L. Bonelli, and S. Felletti, 17–32. College.

[CR38] Oswald, S., and M. Lewiński. 2014. Pragmatics, cognitive heuristics and the straw man fallacy. In *Rhetoric & Cognition Theoretical perspectives and persuasive strategies*, ed. T. Herman and S. Oswald, 313–343. Peter Lang.

[CR39] Petty, R.E., J.T. Cacioppo, and R. Goldman. 1981. Personal involvement as a determinant of argument-based persuasion. *Journal of Personality and Social Psychology* 41 (5): 847–855. 10.1037/0022-3514.41.5.847.

[CR40] Pruś, J., and P. Sikora. 2023. The dialectical principle of charity: A procedure for a critical discussion. *Argumentation* 37: 577–600. 10.1007/s10503-023-09615-8.

[CR41] Rains, S.A., T.R. Levine, and R. Weber. 2018. Sixty years of quantitative communication research summarized: Lessons from 149 meta-analyses. *Annals of the International Communication Association* 42 (2): 105–124. 10.1080/23808985.2018.1446350.

[CR42] Rapoport, A. 1960. *Fights, Games, and Debates*. The University of Michigan Press.

[CR43] Rapoport, A. 1961. Three modes of conflict. *Management Science* 7(3):210–218. 10.1287/mnsc.7.3.210

[CR16] R Core Team. 2024. *R: A Language and Environment for Statistical Computing* [Computer software]. https://www.R-project.org/

[CR44] Rubin, R.B. 2004. Source Credibility Scale—McCroskey. In *Communication Research Measures: A Sourcebook*, ed. R.B. Rubin, P. Palmgreen, and H.E. Sypher, 332–339. Lawrence Erlbaum Associates.

[CR45] Schubert, S., and L. Caviola. 2024. *Effective Altruism and the Human Mind: The Clash Between Impact and Intuition*. Oxford University Press. 10.1093/oso/9780197757376.001.0001.

[CR46] Schumann, J. 2022. Do people perceive the disagreement in straw man fallacies? An experimental investigation. *Languages* 7(2):111. 10.3390/languages7020111

[CR47] Schumann, J., S. Zufferey, and S. Oswald. 2019. What makes a straw man acceptable? Three experiments assessing linguistic factors. *Journal of Pragmatics* 141:1–15. 10.1016/j.pragma.2018.12.009

[CR48] Schumann, J., S. Zufferey, and S. Oswald. 2021. The linguistic formulation of fallacies matters: The case of causal connectives. *Argumentation* 35:361–388. 10.1007/s10503-020-09540-0

[CR49] Sperber, D., and D. Wilson. 1995. *Relevance: Communication and cognition*. 2nd ed. Blackwell Publishers.

[CR50] Sperber, D., F. Clément, C. Heintz, O. Mascaro, H. Mercier, G. Origgi, and D. Wilson. 2010. Epistemic vigilance. *Mind & Language* 25(4):359–393. 10.1111/j.1468-0017.2010.01394.x

[CR51] Tuppen, C.J.S. 1974. Dimensions of communicator credibility: An oblique solution. *Speech Monographs* 41 (3): 253–260. 10.1080/03637757409375844.

[CR53] van Eemeren, F.H., and P. Houtlosser. 1999. Delivering the Goods in Critical Discussion. In *Proceedings of the Fourth International Conference of the International Society for the Study of Argumentation*, ed. J.A. Blair and C.A. Willard, 168–167. Sic Sat.

[CR52] van Eemeren, F.H., and R. Grootendorst. 2004. *A Systematic Theory of Argumentation: The pragma-dialectical approach*. Cambridge University Press.

[CR54] van Eemeren, F.H., B. Garssen, and B. Meuffels. 2009. *Fallacies and Judgments of Reasonableness*, vol. Vol. 16. Springer Netherlands. 10.1007/978-90-481-2614-9.

[CR55] van Eemeren, F.H., B. Garssen, and B. Meuffels. 2012. Effectiveness through reasonableness preliminary steps to pragma-dialectical effectiveness research. *Argumentation* 26: 33–53. 10.1007/s10503-011-9234-7.

[CR56] van Emeren, F.H., R. Grootendorst, and A. Francisca Snoeck Henkemans. 2002. *Argumentation: Analysis, evaluation, presentation*. Lawrence Earlbaum Associates.

[CR57] Walton, D. 1996. The straw man fallacy. In *Logic and Argumentation*, ed. J. van Benthem, F. van Eemeren, R. Grootendorst, and F. Veltman, 115–128. Amsterdam: Royal Netherland Academy of Arts and Sciences.

[CR58] Weber, R., and L. Popova. 2012. Testing equivalence in communication research: Theory and application. *Communication Methods and Measures* 6(3):190–213. 10.1080/19312458.2012.703834

[CR59] Yalch, R.F., and R. Elmore-Yalch. 1984. The effect of numbers on the route to persuasion. *Journal of Consumer Research* 11 (1): 522–527. 10.1086/208988.

[CR9] Younis, R. 2026. Data and materials: (Mis)representing the opposition and rhetorical Success. 10.17605/OSF.IO/CK83Z .

